# Mild *Staphylococcus aureus* Skin Infection Improves the Course of Subsequent Endogenous *S*. *aureus* Bacteremia in Mice

**DOI:** 10.1371/journal.pone.0129150

**Published:** 2015-06-10

**Authors:** Sanne van den Berg, Corné P. de Vogel, Alex van Belkum, Irma A. J. M. Bakker-Woudenberg

**Affiliations:** 1 Department of Medical Microbiology and Infectious Diseases, Erasmus University Medical Center, Rotterdam, the Netherlands; 2 bioMérieux, Microbiology R&D, La Balme les Grottes, France; Montana State University, UNITED STATES

## Abstract

*Staphylococcus aureus* carriers with *S*. *aureus* bacteremia may have a reduced mortality risk compared to non-carriers. A role for the immune system is suggested. Here, we study in mice the effect of mild *S*. *aureus* skin infection prior to endogenous or exogenous *S*. *aureus* bacteremia, and evaluate protection in relation to anti-staphylococcal antibody levels. Skin infections once or twice by a clinical *S*. *aureus* isolate (isolate P) or *S*. *aureus* strain 8325-4 were induced in mice free of *S*. *aureus* and anti-staphylococcal antibodies. Five weeks later, immunoglobulin G (IgG) levels in blood against 25 *S*. *aureus* antigens were determined, and LD50 or LD100 bacteremia caused by *S*. *aureus* isolate P was induced. *S*. *aureus* skin infections led to elevated levels of anti-staphylococcal IgG in blood. One skin infection improved the course of subsequent severe endogenous bacteremia only. A second skin infection further improved animal survival rate, which was associated with increased pre-bacteremia IgG levels against Efb, IsaA, LukD, LukE, Nuc, PrsA and WTA. In conclusion, *S*. *aureus* isolate P skin infection in mice reduces the severity of subsequent endogenous *S*. *aureus* bacteremia only. Although cellular immune effects cannot be rules out, anti-staphylococcal IgG against specified antigens may contribute to this effect.

## Introduction

About 20% of the healthy human population persistently carries *Staphylococcus aureus* in their nose [1–3]. Although carriage of *S*. *aureus* is usually asymptomatic, this bacterial species can also cause infections. These include skin and soft tissue infections such as furunculosis, and also life-threatening invasive diseases such as pneumonia and bacteremia [4]. Nasal carriage of *S*. *aureus* is a major risk factor for the development of surgical site infections caused by *S*. *aureus* [5–9]. Moreover, Wertheim et al. [10] suggested that carriers have a three-fold higher risk than non-carriers of acquiring hospital-associated *S*. *aureus* bacteremia, while the mortality risk in carriers with bacteremia might be lower [10][10].

In spite of the higher risk of acquiring nosocomial *S*. *aureus* bacteremia in *S*. *aureus* carriers, the risk of death due to bacteremia might be lower once carriers acquire bacteremia. An explanation for this has not yet been provided, although a role for the immune system has been proposed. More than 80% of health care-associated *S*. *aureus* infections are caused by an endogenous strain [10, 11]. This suggests that because of long-term exposure to the colonizing *S*. *aureus* strain, carriers may have developed antibodies or cellular immune responses that protect against endogenous bacteremia-related death. Non-carriers may have developed humoral responses that protect against colonization more than against invasive disease. Several studies have been conducted comparing anti-staphylococcal antibody levels in carriers and non-carriers. Carriers show higher immunoglobulin G (IgG) levels than non-carriers against toxic shock syndrome toxin 1 (TSST-1), staphylococcal enterotoxin A (SEA) [12] and the factor effecting methicillin resistance (FmtB) [13]. In contrast, compared to carriers, IgG levels in non-carriers are significantly higher against alpha toxin, major autolysin (Atl), iron-responsive surface determinant A and H (IsdA and IsdH), immunodominant staphylococcal antigen A (IsaA) [13], extracellular adherence protein (Eap), haptoglobin-hemoglobin binding protein A (HarA), and clumping factor B (ClfB) [14]. In addition to these descriptive studies, the prospective clinical study of Kolata et al. [15] also suggested a contribution of antibodies against the colonizing *S*. *aureus* strain in the improvement of the course and outcome of *S*. *aureus* bacteremia. In this study, *S*. *aureus* carriers who developed endogenous *S*. *aureus* bacteremia showed a stronger and broader pre-bacteremia IgG response to their own invasive, endogenous *S*. *aureus* strain compared to non-carriers, who develop an exogenous *S*. *aureus* bacteremia. Recently, Montgomery et al. [16] showed in an experimental study in mice that *S*. *aureus* skin and soft tissue infection (SSTI) protects against secondary endogenous SSTI. This protection was mediated by antibody and interleukin (IL) 17A and inhibited by interferon (IFN) γ. Conclusions regarding the antigen-specificity of these antibodies were not drawn. Their observation suggests, in addition to a role of humoral immunity, a protective role of cellular immunity. Protection against exogenous infection was not studied.

In humans, conclusive studies on the exact influence of *S*. *aureus* carriage and/or *S*. *aureus* exposure and the role of humoral and/or cellular immunity on the course and outcome of subsequent endogenous or exogenous *S*. *aureus* infection are difficult as both carriers and non-carriers harbor a diversity of anti-staphylococcal antibodies. In non-carriers, these antibodies may be induced by *S*. *aureus* carriage or (sub-)clinical infection in the past. Studies in mice initially free of *S*. *aureus* and anti-staphylococcal antibodies may provide further insight.

In the present study in mice, we investigated whether the course of *S*. *aureus* bacteremia is influenced by prior *S*. *aureus* exposure and whether this is dependent on the *S*. *aureus* strain (endogenous or exogenous) causing the initial exposure. For this purpose, a mouse model of mild *S*. *aureus* skin infection once or twice was established. We focused in this study on humoral immunity only, by analyzing the pre-bacteremia IgG levels against a broad panel of 25 *S*. *aureus* antigens following skin infection, and we assessed whether improvement in the course of *S*. *aureus* bacteremia was associated with pre-bacteremia IgG levels.

## Materials and Methods

### Bacteria

Bacterial strains used were a clinical *S*. *aureus* isolate and *S*. *aureus* strain 8325–4, a well-characterized laboratory strain (MSSA, ST8) [17]. *S*. *aureus* isolate P (gift from G. Buist, University of Groningen, University Medical Center Groningen, Groningen, the Netherlands) is a clinical isolate recovered from blood of a septic patient and was previously described and analyzed by proteomics by Ziebandt et al. (community-acquired MSSA, ST7, *agr*-type 1) [18]. Staphylococci were grown overnight at 35°C on Colombia III blood agar (Becton Dickinson, Breda, the Netherlands). Cultures of *S*. *aureus*, grown in Brain Heart Infusion broth (Becton Dickinson, Breda, the Netherlands) until OD_560_ ~ 1.0, were stored with 5% glycerol at -80°C. For infection, a suspension of staphylococci was defrosted and centrifuged for 10 minutes at 14,000 x *g*. The *S*. *aureus* pellet was resuspended in saline, and diluted to obtain the desired inoculum.

### Animals

Specified opportunistic pathogen-free (SOPF) female BALB/cBYJ mice were obtained from Charles River (Saint-Germain-sur-l’Arbresle, France). These *S*. *aureus*-free animals were 11–13 weeks old on the day of infection, and were given food and water *ad libitum*. Before each experiment, one mouse per group was sacrificed to confirm the *S*. *aureus*-free status in terms of cultures of fresh fecal and nasal microbiota and the absence of anti-staphylococcal IgG levels in blood (see below). The animal experimental protocols adhered to the rules laid down in the Dutch Animal Experimentation Act and the EU Animal Directive 2010/63/EU, and the Institutional Animal Care and Use Committee of the Erasmus University Medical Centre Rotterdam approved the present protocols (permit **number**: EMC2415).

### Model of *S*. *aureus* skin infection

The method of induction of *S*. *aureus* skin infection in mice was adapted from Brown et al. [19]. In short, the lower back of the mice was shaved and cleaned with 70% ethanol under general anesthesia after using a mixture of medetomidine (Sedator^®^, 0.5 mg/kg; Eurovet Animal Health, Bladel, the Netherlands), midazolam (Midazolam, 5 mg/kg; Actavis, Baarn, the Netherlands) and fentanyl (Fentanyl, 0.05 mg/kg; Hameln Pharmaceuticals, Hameln, Germany). *S*. *aureus* isolate P (3–6 x 10^7^ CFU) or *S*. *aureus* 8325–4 (5–10 x 10^7^ CFU) was injected intradermally (50 μL) (n = 10 per group). For placebo skin infection, mice received saline. Anesthesia was antagonized to quickly awaken the mice using a mixture of atipamezole (Antisedan^®^, 2.5 mg/kg; Orion Corporation, Espoo, Finland), flumazenil (Flumazenil, 0.5 mg/kg; Pharmachemie, Haarlem, the Netherlands) and naloxon (Naloxon, 1.2 mg/kg; Orpha-Devel Handels und Vertriebs, Purkersdorf, Germany). Anesthetic and antagonistic agents were administered intraperitoneally, in a total volume of 175 and 250 μL, respectively. Mouse body weight was assessed three times a week. Two and five weeks after intradermal *S*. *aureus* inoculation, blood was withdrawn from the tail artery to determine anti-staphylococcal IgG levels in serum. Blood was collected in a Microvette^®^ CB300 tube (Sarstedt, Etten-Leur, the Netherlands) and sera were prepared and stored at -80°C. At week five, mice were sacrificed by CO_2_ exposure. Presence of *S*. *aureus* in intestines and nasopharynx five weeks after intradermal *S*. *aureus* inoculation was determined by culturing fresh feces and nasopharyngeal lavage in phenol-red mannitol salt broth (PHMB; Becton Dickinson, Breda, the Netherlands) at 35°C for 7 days. Nasopharyngeal lavage was performed by flushing the nares with 5 mL sterile phosphate buffered saline + 0.4% Tween 20 (Sigma-Aldrich, Zwijndrecht, the Netherlands). When PHMB turned yellow, this was subcultured overnight at 35°C on Colombia III blood agar. *S*. *aureus* was identified based on colony morphology and Slidex Staph Plus agglutination testing (bioMérieux, Marcy l’Etoile, France). Multi-locus variable number of tandem repeat analysis (MLVA) of SIRU01, SIRU07, SIRU13 and SIRU15 [20] was used at regular intervals to verify that isolated *S*. *aureus* strains were the same as the strain used for infection of mice.

### Model of *S*. *aureus* bacteremia

Bacteremia was induced by inoculation of 100 μL of *S*. *aureus* isolate P into the tail vein. A *S*. *aureus* inoculum at the 50% lethal dose (LD50; 1–2 x 10^5^ CFU) or at the 100% lethal dose (LD100; 5–8 x 10^5^ CFU) was used for establishment bacteremia. Clinical signs of illness in each mouse were evaluated twice daily during the experiment to minimize suffering as described before [21]. Mice were scored -1 directly after bacterial inoculation. Mice with bad fur were scored -2. Mice with bad fur and hunched back were scored -3. Mice with bad fur and hunched back and that showed instability were scored -4. These mice showed severe signs of illness and were euthanized by CO_2_ exposure. Euthanized mice were considered as deaths, as pilot experiments showed that mice with severe signs of illness die before the next time point. Animal survival rate over 14 days after infection was monitored. At day 28, *S*. *aureus* load in blood and organs of surviving animals was determined to investigate whether surviving mice next to survival also showed improved *S*. *aureus* clearance. The mice were sacrificed by CO_2_ exposure. A blood sample was taken via a transcutaneous cardiac puncture and collected in a vial containing Lithium Heparin (Sarstedt, Etten-Leur, the Netherlands). The lungs, spleen, liver, and kidneys were removed aseptically and homogenized (Polytron, Kinematica, Luzern, Switzerland) in 2 mL of saline for 10 seconds at 30,000 rpm at room temperature. Undiluted homogenate suspensions and blood as well as 10-fold serial dilutions in saline were plated onto Colombia III blood agar. After incubation overnight at 35°C, colonies were counted.

### Experimental set-up to study the course of *S*. *aureus* bacteremia in relation to prior *S*. *aureus* skin infection

**Fig 1:**
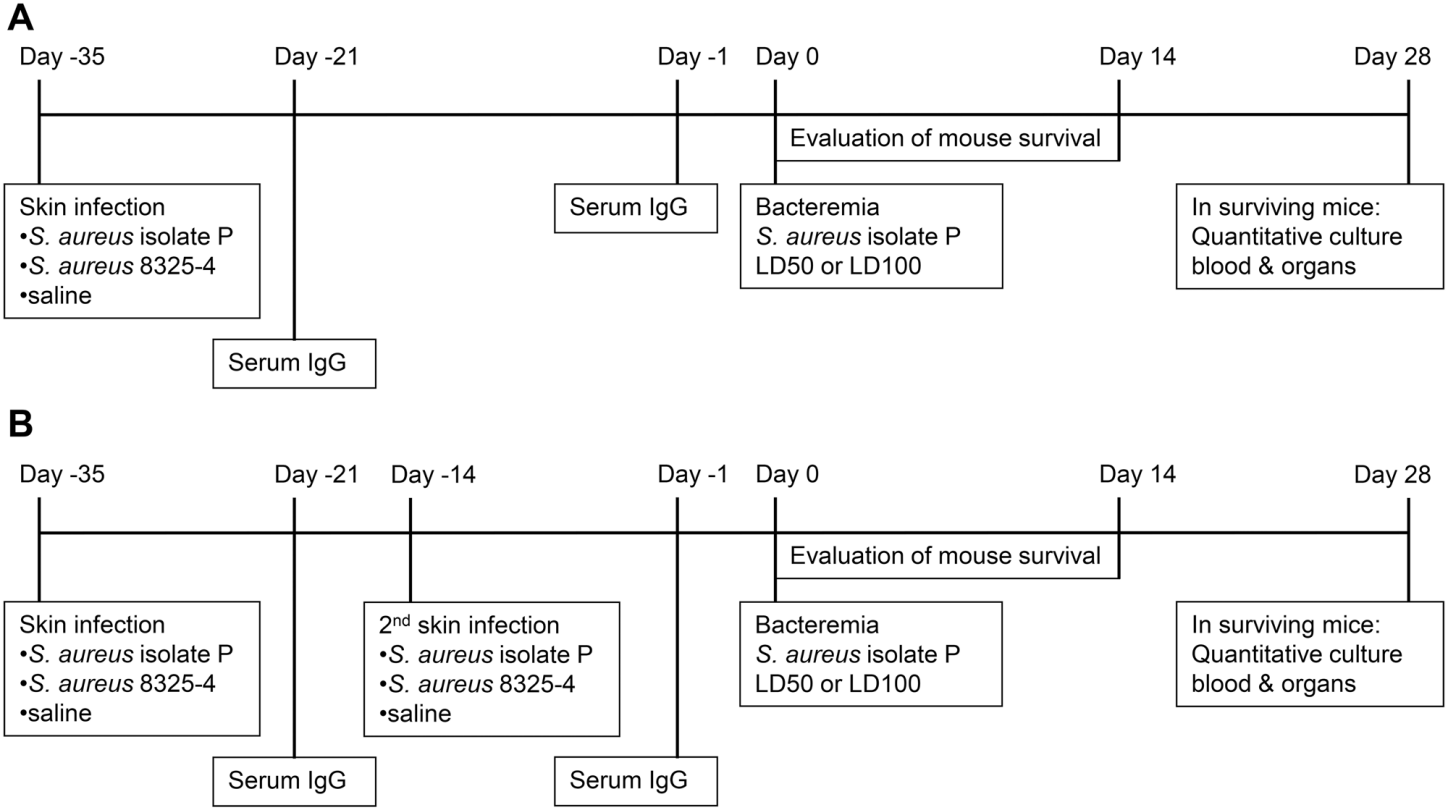
Experimental set-up for studying the influence of *S*. *aureus* skin infection on subsequent bacteremia. *A*. Skin infection once at 35 days before *S*. *aureus* bacteremia. *B*. Skin infection twice at 35 days and 14 days before *S*. *aureus* bacteremia.

Experimental set-up is shown in Fig 1. Skin infection with *S*. *aureus* isolate P or with *S*. *aureus* 8325–4 (n = 15 per group) was induced 35 days before induction of bacteremia. Controls received intradermal inoculation of saline. In case of skin infection twice, three weeks after the first skin infection, a second skin infection using the same *S*. *aureus* strain was applied, near the inoculation site of the first skin infection. Bacteremia caused by *S*. *aureus* isolate P was always established at five weeks after the first skin infection, when mice were 16–18 weeks old (adults).

### Quantification of serum anti-staphylococcal IgG levels

Serum IgG levels against the following antigens were semi-quantified: alpha toxin; clumping factors A and B (ClfA and ClfB); extracellular fibrinogen-binding protein (Efb); ESAT-6-like protein (EsxA) and CFP-10-like protein (EsxB); formyl peptide receptor-like1 inhibitory protein (FLIPr); fibronectin-binding protein A (FnbpA); immunodominant staphylococcal antigen A (IsaA); iron-responsive surface determinants A and H (IsdA and IsdH); lipase; leukocidins D and E (LukD and LukE); peptidoglycan hydrolase (LytM); endonuclease (Nuc); peptidoglycan (PG); a parvulin-type peptidyl-prolyl *cis/trans* isomerase (PrsA); hypothetical protein SA0104; serine-aspartate dipeptide repeat protein D (SdrD); staphylococcal superantigen-like proteins 1, 3, 5, and 10 (SSL1, SSL3, SSL5, and SSL10); and wall teichoic acid (WTA). Genes encoding these *S*. *aureus* antigens were all present in both *S*. *aureus* isolate P and *S*. *aureus* 8325–4.

Alpha toxin, LukD, and LukE were prepared as described previously [22]. ClfB, FnbpA, IsdA, IsdH, and SdrD were expressed and purified as described previously [12]. The constructs were kindly provided by T. Foster (Trinity College, Dublin, Ireland). All other antigens were kindly provided by other research groups, as indicated in the Acknowledgements.

IgG levels were semi-quantified simultaneously in multiplex assays using a bead-based flow cytometry technique (xMap; Luminex Corporation, Austin, TX). Methods have been described elsewhere [23–26]. Tests were performed in independent duplicates, and the median fluorescence intensity (MFI) values, reflecting semi-quantitative antibody levels, were averaged. In each experiment, control beads (no antigen coupled) were included to determine nonspecific binding. In case of nonspecific binding, the nonspecific MFI values were subtracted from the antigen-specific values.

Serum samples from mice with skin infection caused by *S*. *aureus* isolate P or *S*. *aureus* 8325–4 (n = 10 per group), and from mice with placebo skin infection were analyzed. Sera from three non-infected mice were used as negative controls.

### Statistical analysis

The Mann-Whitney *U* test was used to compare median differences in anti-staphylococcal IgG levels in different groups. The Wilcoxon Signed Rank test was used to compare anti-staphylococcal IgG levels in paired samples. In individual mice, high IgG levels against an antigen were not correlated with high IgG levels against other antigens, or vice versa. The Bonferroni correction was applied to correct for multiple testing. As a result, *P*-values < 0.002 were considered to be statistically significant. These statistical analyses were performed using the Statistical Package of Social Sciences version 17.0 for Windows (SPSS Inc., Chicago, IL).

The Fisher’s exact test was used to compare differences in *S*. *aureus* colonization status. The log rank test was used to determine statistical differences in animal survival rate between groups. Differences were considered statistically significant when 2-sided *P*-values were < 0.05. GraphPad Prism 5 for Windows (GraphPad Software Inc., La Jolla, CA) was used for these statistical analyses.

## Results

### *S*. *aureus* skin infection

Mice infected with *S*. *aureus* isolate P or *S*. *aureus* 8325–4 developed a scab (area of ~1.5 cm^2^) at the inoculation site within 1 week. Body weight loss was only minor (maximum of 8% at week 1 after intradermal inoculation) and mice appeared healthy. Five weeks after infection, when *S*. *aureus* bacteremia was induced, the skin had healed and body weight was restored. *S*. *aureus* cultures from the inoculation site were always negative at week 5 after skin infection. However, the intestines of mice with skin infection once or twice, caused by *S*. *aureus* isolate P or *S*. *aureus* 8325–4, were always culture positive for the infecting *S*. *aureus* strain. Nasopharynges were not always culture positive: *S*. *aureus* was cultured from mice after *S*. *aureus* isolate P skin infection once (3 of 10 mice) or twice (5 of 10 mice), and after *S*. *aureus* 8325–4 skin infection once (1 of 10 mice) or twice (4 of 10 mice). No significant differences in number of nasopharynx culture positive mice were observed.

### Anti-staphylococcal IgG levels following skin infection

Serum anti-staphylococcal IgG levels against a broad panel of 25 staphylococcal antigens were assessed in mice with *S*. *aureus* isolate P skin infection once or twice (Table 1) or with *S*. *aureus* 8325–4 skin infection once or twice (Table 2) (n = 10 per group). Levels were assessed at two and five weeks after the first skin infection. Antigens included immune modulators, superantigen like proteins, MSCRAMMs (microbial surface components recognizing adhesive matrix molecules), toxins, and household antigens. Before infection (week 0), IgG levels were at background values. In mice with skin infection once caused by *S*. *aureus* isolate P or *S*. *aureus* 8325–4, IgG levels against a number of *S*. *aureus* antigens were observed at week 2 and 5 after infection, but these did not change significantly over time. However, following the second skin infection with *S*. *aureus* isolate P, the IgG levels against IsaA, Nuc, PrsA, and WTA were significantly elevated at week 5 (*P* < 0.002), whereas the second skin infection with *S*. *aureus* 8325–4 resulted in a significant rise in IgG levels at week 5 against Efb, IsaA, and IsdA (*P* < 0.002).

**Table 1:** *S*. *aureus* isolate P skin infection. Median fluorescence intensity (MFI) values reflecting levels of antigen-specific IgG against 25 *S*. *aureus* antigens in sera from mice (n = 10 per group) before skin infection (week 0), after skin infection once (week 2 and week 5), and after skin infection twice (week 5 after first skin infection).

		Skin infection once		Skin infection twice				
Antigen	Median MFI week 0 (range)	Median MFI week 2 (range)	Median MFI week 5 (range)	Median MFI week 5 (range)	*P*-value (skin infection once) week 2 vs week 0^*a*^	*P*-value (skin infection once) week 5 vs week 0^*a*^	*P*-value (skin infection once) week 5 vs week 2^*b*^	*P*-value (week 5) skin infection twice vs once^*a*^
alpha toxin	0 (0–1)	644 (203–1712)	5693 (4402–6704)	3994 (3381–5922)	0.011	0.011	0.005	0.007
ClfA	0 (0–0)	0 (0–0)	0 (0–4)	0 (0–5)	1.000	0.584	0.317	0.304
ClfB	0 (0–0)	0 (0–0)	0 (0–14)	0 (0–4)	1.000	0.584	0.317	0.358
Efb	3 (1–6)	4 (2–8)	11 (7–1536)	143 (33–1590)	0.611	0.011	0.005	0.070
EsxA	5 (5–6)	5 (5–6)	6 (4–8)	5 (3–8)	0.661	0.495	0.313	0.569
EsxB	3 (3–4)	4 (3–24)	5 (3–46)	4 (2–177)	0.551	0.062	0.028	0.119
FLIPr	0 (0–0)	0 (0–0)	0 (0–0)	0 (0–44)	1.000	1.000	1.000	0.068
FnbpA	0 (0–0)	0 (0–3)	0 (0–14)	0 (0–0)	0.584	0.584	0.317	0.317
IsaA	5 (4–6)	12 (6–1504)	8 (5–1509)	3885 (247–8090)	0.014	0.034	0.386	0.001 *
IsdA	103 (3–106)	77 (0–108)	30 (0–94)	70 (0–432)	0.498	0.236	0.012	0.072
IsdH	63 (27–68)	38 (21–61)	36 (11–50)	41 (20–1065)	0.236	0.236	0.475	0.257
lipase	6 (6–6)	9 (8–14)	8 (7–20)	80 (9–8188)	0.011	0.011	0.959	0.002
LukD	0 (0–0)	0 (0–0)	103 (1–1986)	1595 (34–5177)	0.584	0.011	0.005	0.019
LukE	2 (0–2)	235 (13–777)	4470 (2110–6930)	6487 (4921–7596)	0.011	0.011	0.005	0.023
LytM	1 (1–3)	2 (0–5)	2 (1–3)	4 (2–66)	0.865	0.441	0.918	0.015
Nuc	2 (1–3)	6 (0–270)	405 (2–3999)	4554 (930–5686)	0.128	0.028	0.013	0.001 *
PG	6 (4–6)	6 (5–6)	6 (5–7)	6 (5–7)	0.302	0.395	0.777	0.223
PrsA	32 (28–33)	405 (194–900)	3282 (1728–4490)	4568 (3592–5838)	0.011	0.011	0.005	0.0019 *
SA0104	5 (4–6)	6 (4–19)	6 (4–34)	6 (5–9)	0.125	0.106	0.574	0.939
SdrD	0 (0–0)	0 (0–10)	0 (0–22)	0 (0–5)	0.584	0.584	0.317	0.942
SSL1	0 (0–0)	0 (0–50)	1 (0–63)	21 (0–34)	0.215	0.148	0.500	0.292
SSL3	0 (0–0)	0 (0–0)	0 (0–0)	0 (0–0)	1.000	1.000	1.000	1.000
SSL5	0 (0–0)	0 (0–2)	3 (0–274)	0 (0–20)	0.584	0.098	0.028	0.033
SSL10	0 (0–0)	0 (0–2)	1 (0–12)	0 (0–2)	0.147	0.098	0.324	0.606
WTA	1 (0–1)	1 (0–2)	4 (2–10)	22 (4–78)	0.283	0.011	0.008	0.0019 *

^a^ Mann-Whitney U test

^b^ Wilcoxon signed ranks test

* P < 0.002

**Table 2:** *S*. *aureus* 8325–4 skin infection. Median fluorescence intensity (MFI) values reflecting levels of antigen-specific IgG against 25 *S*. *aureus* antigens in sera from mice (n = 10 per group) before skin infection (week 0), after skin infection once (week 2 and week 5), and after skin infection twice (week 5 after first skin infection).

		Skin infection once		Skin infection twice				
Antigen	Median MFI week 0 (range)	Median MFI week 2 (range)	Median MFI week 5 (range)	Median MFI week 5 (range)	*P*-value (skin infection once) week 2 vs week 0^*a*^	*P*-value (skin infection once) week 5 vs week 0^*a*^	*P*-value (skin infection once) week 5 vs week 2^*b*^	*P*-value (week 5) skin infection twice vs once^*a*^
alpha toxin	0 (0–1)	1129 (354–2341)	5545 (2110–6932)	4401 (2878–6605)	0.011	0.011	0.005	0.258
ClfA	0 (0–0)	0 (0–0)	0 (0–1)	0 (0–0)	1.000	0.584	0.317	0.317
ClfB	0 (0–0)	0 (0–0)	0 (0–0)	0 (0–2)	1.000	1.000	1.000	0.317
Efb	3 (1–6)	4 (2–8)	8 (6–12)	24 (15–80)	0.445	0.017	0.009	0.0002 *
EsxA	5 (5–6)	5 (3–6)	6 (4–6)	6 (5–6)	0.865	0.670	0.366	0.335
EsxB	3 (3–4)	3 (3–12)	4 (3–6)	4 (2–39)	0.733	0.351	0.483	0.731
FLIPr	0 (0–0)	0 (0–0)	0 (0–0)	0 (0–0)	1.000	1.000	1.000	1.000
FnbpA	0 (0–0)	0 (0–0)	0 (0–0)	0 (0–2)	1.000	1.000	1.000	0.147
IsaA	5 (4–6)	7 (4–61)	6 (5–166)	186 (12–6838)	0.235	0.089	0.918	0.001 *
IsdA	103 (3–106)	54 (0–129)	82 (0–221)	525 (120–1145)	0.397	0.612	0.161	0.001 *
IsdH	63 (27–68)	34 (28–60)	36 (14–53)	37 (19–61)	0.398	0.237	0.359	0.650
lipase	6 (6–6)	12 (5–166)	141 (8–1992)	405 (8–6865)	0.011	0.011	0.008	0.364
LukD	0 (0–0)	0 (0–61)	7 (0–112)	11 (2–57)	0.304	0.037	0.012	0.325
LukE	2 (0–2)	5 (0–345)	57 (5–1008)	118 (7–1743)	0.034	0.011	0.022	0.364
LytM	1 (1–3)	2 (1–3)	2 (1–3)	3 (1–22)	0.492	0.293	0.429	0.333
Nuc	2 (1–3)	1 (0–13)	2 (0–15)	11 (1–1646)	0.497	0.670	0.130	0.031
PG	6 (4–6)	6 (5–6)	6 (4–7)	6 (5–7)	0.664	0.389	0.589	0.373
PrsA	32 (28–33)	190 (43–303)	1074 (153–2502)	3167 (264–4967)	0.011	0.011	0.005	0.034
SA0104	5 (4–6)	6 (5–8)	5 (4–8)	7 (4–23)	0.034	0.391	0.087	0.030
SdrD	0 (0–0)	0 (0–0)	0 (0–0)	0 (0–3)	1.000	1.000	1.000	0.317
SSL1	0 (0–0)	1 (0–93)	45 (0–3834)	41 (1–4112)	0.148	0.062	0.043	0.545
SSL3	0 (0–0)	0 (0–0)	0 (0–0)	0 (0–0)	1.000	1.000	1.000	1.000
SSL5	0 (0–0)	0 (0–0)	0 (0–0)	0 (0–5)	1.000	1.000	1.000	0.317
SSL10	0 (0–0)	0 (0–5)	0 (0–6)	1 (0–5)	0.215	0.148	0.564	0.209
WTA	1 (0–1)	1 (0–2)	2 (0–7)	3 (1–12)	0.122	0.383	0.443	0.068

^a^ Mann-Whitney U test

^b^ Wilcoxon signed ranks test

* *P* < 0.002

### Course of *S*. *aureus* bacteremia in mice with prior skin infection

Bacteremia was always induced by *S*. *aureus* isolate P irrespective of the strain used for skin infection. Animal survival rate after bacteremia was monitored over a 14-day evaluation period. After this time point, no further changes in animal survival were observed (data not shown).

**Fig 2:**
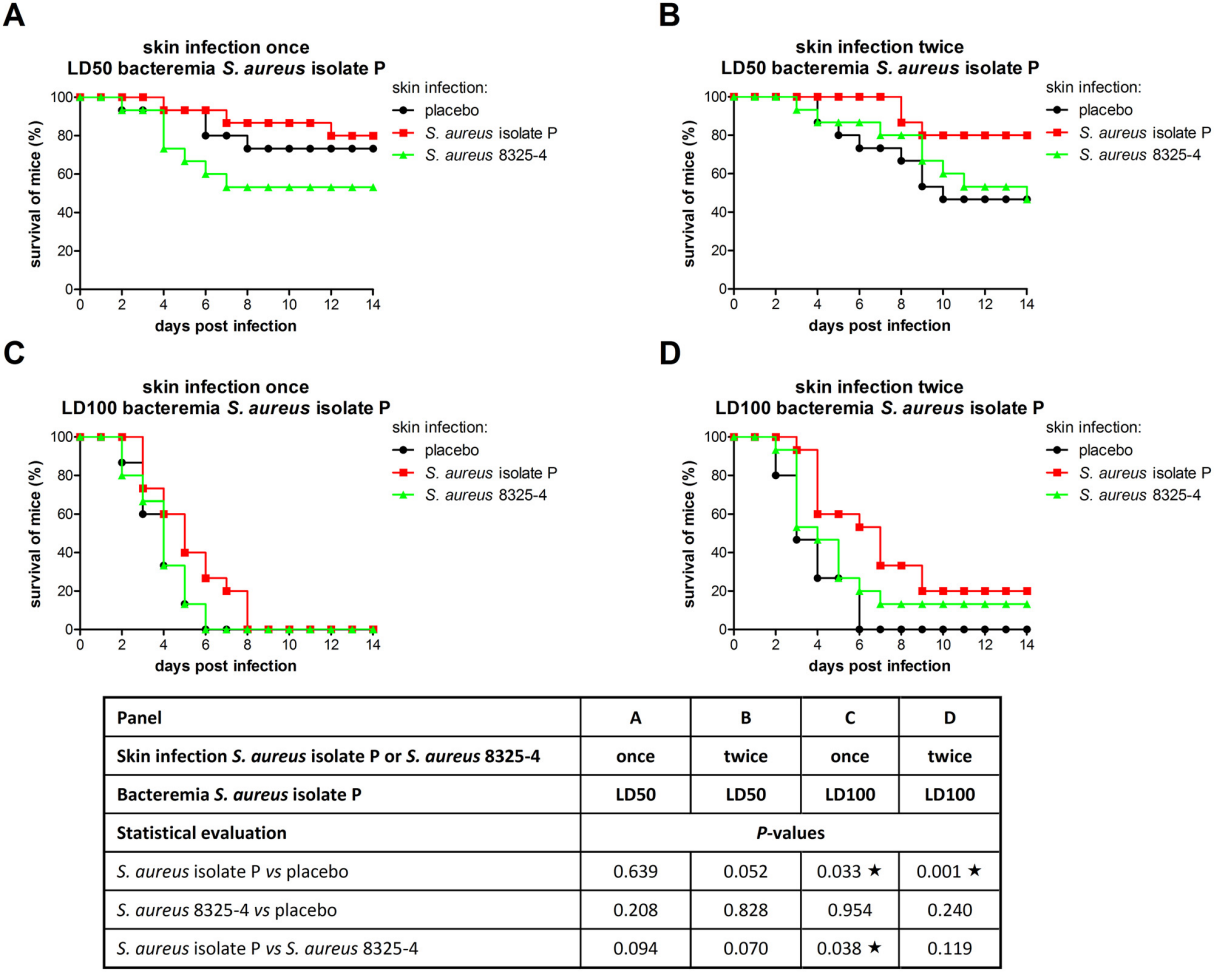
Survival rate in mice with *S*. *aureus* bacteremia. Skin infection prior to *S*. *aureus* bacteremia was induced once or twice by *S*. *aureus* isolate P (red squares) or *S*. *aureus* 8325–4 (green triangles); placebo skin infection (black circles). LD50 or LD100 bacteremia was induced by *S*. *aureus* isolate P. Animal survival rate in different groups (n = 15 per group) was compared using the log-rank test, and *P*-values are indicated in the table. Stars indicate statistically significant differences (*P* < 0.05).

The course of LD50 *S*. *aureus* bacteremia in mice with prior skin infection is shown in Fig 2A and 2B (n = 15 per group). In mice with placebo skin infection, survival of bacteremic mice declined gradually, until 47–73% at day 14. Survival rates of mice with placebo skin infection once or twice were comparable. After skin infection once or twice with *S*. *aureus* isolate P or *S*. *aureus* 8325–4, animal survival rate of bacteremic mice was not significantly improved compared to placebo skin infection.

The course of LD100 *S*. *aureus* bacteremia in mice with prior skin infection is shown in Fig 2C and 2D (n = 15 per group). In mice with placebo skin infection, survival of bacteremic mice declined gradually, and at day 6 all mice had died. Survival rates of mice with placebo skin infection once or twice were comparable. An increased animal survival rate of bacteremic mice was observed only after prior skin infection with *S*. *aureus* isolate P. The time to death of bacteremic mice was increased after skin infection once (*P* = 0.033), and was further prolonged after the second skin infection (*P* = 0.001). Mouse survival at day 14 was increased from 0% to 20%. Skin infection once or twice with *S*. *aureus* 8325–4 had no effect on the course of bacteremia.

In mice that survived LD50 bacteremia (Fig 2A and 2B) or LD100 bacteremia (Fig 2D), *S*. *aureus* was never cultured from blood, lungs, spleen, and liver at day 28. In 17–60% of surviving mice, cultures from kidneys were *S*. *aureus* positive, ranging from 1 x 10^1^–2 x 10^7^ CFU per two kidneys, but differences in number of *S*. *aureus* positive kidneys between the mice that survived moderate bacteremia were not observed

### Course of *S*. *aureus* bacteremia in association with anti-*S*. *aureus* IgG levels

**Fig 3:**
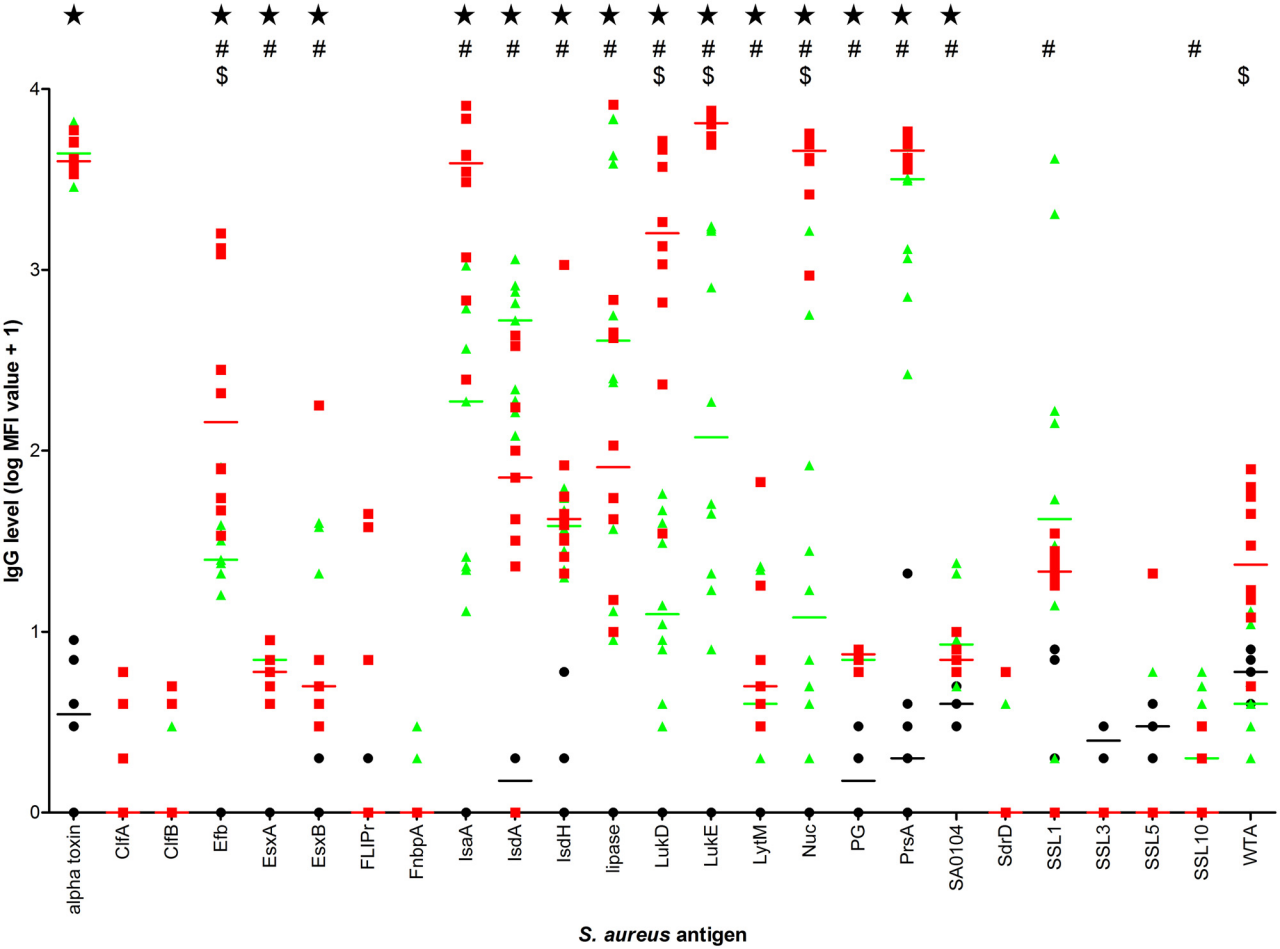
Antigen-specific IgG levels in mice at week 5 after *S*. *aureus* skin infection twice. Median fluorescence intensity (MFI) values reflect levels of IgG against 25 *S*. *aureus* antigens. Skin infections were induced by *S*. *aureus* isolate P (red squares) or *S*. *aureus* 8325–4 (green triangles); placebo skin infection (black circles) (n = 10 per group). Median values are indicated by horizontal lines. Stars indicate significant differences (*P* < 0.002) between mice with *S*. *aureus* isolate P skin infection and mice with placebo skin infection. Number signs indicate significant differences (*P* < 0.002) between mice with *S*. *aureus* 8325–4 skin infection and mice with placebo skin infection. Dollar signs indicate significant differences (*P* < 0.002) between mice with *S*. *aureus* isolate P skin infection and mice with *S*. *aureus* 8325–4 skin infection.

We assessed whether improvement of the course of *S*. *aureus* bacteremia was associated with increased levels of anti-staphylococcal IgG induced by *S*. *aureus* skin infection. To this aim, in mice receiving skin infection twice caused by *S*. *aureus* isolate P or *S*. *aureus* 8325–4 (n = 10 per group), serum IgG levels against a broad panel of 25 staphylococcal antigens at week 5 after skin infection, the day of induction of *S*. *aureus* isolate P bacteremia were compared. IgG levels are shown in Fig 3. In *S*. *aureus* isolate P skin infected mice, IgG levels against alpha toxin, Efb, EsxA, EsxB, IsaA, IsdA, IsdH, lipase, LukD, LukE, LytM, Nuc, PG, PrsA, and SA0104 were significantly higher than in mice with placebo skin infection. In *S*. *aureus* 8325–4 skin infected mice, IgG levels against Efb, EsxA, EsxB, IsaA, IsdA, IsdH, lipase, LukD, LukE, LytM, Nuc, PG, PrsA, SA0104, SSL1 and SSL10 were significantly higher than in mice with placebo skin infection. Comparing both IgG profiles, IgG levels against Efb, LukD, LukE, Nuc, and WTA were significantly higher in *S*. *aureus* isolate P skin infected mice than in *S*. *aureus* 8325–4 skin infected mice.

## Discussion

In the present study, we showed for the first time that mild *S*. *aureus* skin infection improved the course of subsequent endogenous *S*. *aureus* bacteremia. The use of *S*. *aureus*-free mice enabled us to draw conclusions in this respect. In humans, conclusive studies on the exact influence of *S*. *aureus* exposure on the outcome of *S*. *aureus* bacteremia remain difficult, as both carriers and non-carriers have been exposed to *S*. *aureus* [12–15]. Wertheim et al. [10] already showed that carriers may have a decreased risk of death due to *S*. *aureus* bacteremia compared to non-carriers, hypothesizing that anti-staphylococcal antibody levels or components of the cellular immune system that are increased in carriers, induced by prior *S*. *aureus* exposure, may play a role in protection against death due to *S*. *aureus* bacteremia. Our observations in the present study in mice are in line with the suggestions made by Wertheim et al.

We established local *S*. *aureus* skin infection caused by either *S*. *aureus* isolate P, a clinical sepsis isolate [18], or *S*. *aureus* 8325–4, a frequently used sequenced strain [17]. Subsequent bacteremia was always induced by *S*. *aureus* isolate P. Skin infection led to *S*. *aureus* colonization in intestines and nasopharynges, and with respect to the humoral immune response, anti-staphylococcal IgG in blood was found. The skin infection described above was mild and transient, simulating a mild *S*. *aureus* infection in humans. We showed that prior *S*. *aureus* skin infection improved the course of LD100 *S*. *aureus* isolate P bacteremia provided that bacteremia was caused by the endogenous *S*. *aureus* strain. This effect was not observed in exogenous bacteremia caused by *S*. *aureus* isolate P when skin infection was induced by *S*. *aureus* 8325–4. While a single prior skin infection resulted in delayed time to death and improved animal survival in our endogenous LD100 *S*. *aureus* bacteremia model, in endogenous LD50 *S*. *aureus* bacteremia prior skin infection did not. This lack of a protective effect in LD50 *S*. *aureus* bacteremia may be related to the relatively small window to detect significant differences in this respect. Whereas in the present study bacteremia was always caused by *S*. *aureus* isolate P, in future experiments it would be informative to study the effects of skin infection on the course and outcome of subsequent *S*. *aureus* 8325–4 bacteremia. *S*. *aureus* 8325–4 showed a very steep inoculum-response curve in the mouse model of bacteremia, and as a consequence unfortunately, we were not able to realize reproducible infection models with LD50 and LD100 inocula.

We also demonstrated that a second skin infection following the first skin infection further enhanced the survival rate in mice with endogenous LD100 *S*. *aureus* bacteremia. Also in the model of endogenous LD50 *S*. *aureus* bacteremia, mortality was reduced, although this difference was only borderline significant (*P* = 0.052), indicating a trend in improvement of survival rate in skin infected mice with subsequent endogenous bacteremia. Interestingly, following the second skin infection, pre-bacteremia serum IgG levels were elevated as well. As improved survival rates were associated with strong increases in pre-bacteremia anti-staphylococcal IgG levels, this suggests that IgG resulting from prior mild skin infection may potentially act protective against antigens determining the outcome of *S*. *aureus* bacteremia. Future studies including successful adoptive transfer of antibody purified from serum of skin infected mice to naive mice prior to *S*. *aureus* bacteremia are necessary to confirm a protective role of pre-bacteremia IgG.

Regarding the pre-bacteremia IgG levels, most striking were the IgG levels against IsaA, Nuc, PrsA, and WTA, which were further elevated following the second skin infection. These IgG levels may contribute to the delayed time to death and improved animal survival in mice with *S*. *aureus* isolate P bacteremia. Next to IgG against these *S*. *aureus* antigens, pre-bacteremia IgG against Efb, LukD, and LukE may also contribute in this respect. These IgG levels were elevated in mice with *S*. *aureus* isolate P skin infection twice and endogenous bacteremia compared to mice with *S*. *aureus* 8325–4 skin infection twice and exogenous bacteremia.

Our observation that *S*. *aureus* isolate P skin infection improved the course of subsequent endogenous *S*. *aureus* bacteremia in mice are in line with those obtained in a recently published experimental study in mice by Montgomery et al. [16]. They studied the effect of *S*. *aureus* SF8300 (USA300) skin infection (SSTI) on a secondary SSTI caused by the same *S*. *aureus* strain. It was shown that skin infection protected against the subsequent skin infection. Protection against exogenous SSTI was not studied. They demonstrated that this protection was mediated by antibody and IL-17A and inhibited by IFN-γ as shown by antibody transfer to naive mice and neutralization of IL-17A or IFN-γ prior to infection, respectively. While in their study IgG levels against Hla and IsdB were assessed, the present study in mice included IgG levels against a broad panel of 25 *S*. *aureus* antigens. Future studies in mice on the effect of immunization targeting IsaA, Nuc, PrsA, WTA, Efb, LukD and LukE prior to the induction of *S*. *aureus* bacteremia may shed further light on the protective role of these anti-staphylococcal IgGs in bacteremia. In addition to these antigens, antibodies against *S*. *aureus* antigens not included in the present study may also be associated with protection against *S*. *aureus* bacteremia. Further insight may be obtained from adoptive antibody transfer studies.

Next to the potential role of pre-bacteremia anti-staphylococcal antibodies, components of cellular immunity are expected to contribute as well to the improvement of the animal survival rate in mice with endogenous *S*. *aureus* bacteremia, as the cellular immune response has a role in *S*. *aureus* infections [16, 27, 28]. Regarding the role of cellular immunity in reduction of *S*. *aureus* bacteremia-related mortality, conclusions cannot be drawn from the present study which was focused on the humoral immune response prior to *S*. *aureus* bacteremia. However Montgomery et al. [16] showed that in their model of SSTI protection against the second skin infection in BALB/c mice was mediated by both antibody and IL-17A. It would be very interesting to investigate the role of IL-17A and other components of cellular immunity in the *S*. *aureus* bacteremia model used in the present study.

In addition to pre-bacteremia immune responses, the consequences of exposure to *S*. *aureus* in the intestines and nasopharynx following *S*. *aureus* skin infection may also contribute to the improvement of the animal survival rate. As differences in number of intestine or nasopharynx culture positive mice were not observed between the groups, no conclusions can be drawn on the immunological relevance of colonization of these sites. As quantitative cultures from the intestinal and nasopharyngeal flora were not performed, definite conclusions on the role of intestinal and nasopharyngeal colonization cannot be drawn, but this merits further investigation.

In conclusion, we show that *S*. *aureus* isolate P skin infection prior to *S*. *aureus* bacteremia improved the outcome of endogenous invasive infection. Improved animal survival rates may be associated with the elevated levels of anti-staphylococcal IgGs against specified antigens. The observation that anti-*S*. *aureus* IgG may contribute to improvement of the course and outcome of endogenous *S*. *aureus* bacteremia opens new perspectives to investigate the protective capacity of active and passive immunization as a non-antibiotic-based treatment regimen in patients with *S*. *aureus* bacteremia. Further studies using other *S*. *aureus* strains are needed to generalize and support our conclusion that *S*. *aureus* skin infection improves the course of subsequent endogenous *S*. *aureus* bacteremia. In this respect, also the role of cellular immunity should be further investigated.
